# Supplementary material for: Phenotypic Variability in Resistance to Anthracnose, White, Brown, and Blight Leaf Spot in Cassava Germplasm
Source: Plants (Basel). 2024 Apr 25;13(9):1187. doi: 10.3390/plants13091187 (PMC11085178; doi:10.3390/plants13091187)
Supplement: Supplementary file 1 [file plants-13-01187-s001.zip › plants-2937395-supplementary.pdf]

Table S1. Variance components for all cassava agronomic attributes.

	$\sigma^2_{C\times L\times Y}$	$\sigma^2_{C\times Y}$	$\sigma^2_{C\times L}$	σ^2_{C}	$\sigma^2_{L\times Y}$	σ^2_{Y}	σ^2_{L}	σ^2
LR	0.061	0	0	0.005	0	0.408	0.024	0.548
DRY	2.261	1.062	0.089	1.106	2.869	2.13	0.356	7.676
DMC	1.554	1.036	0	1.268	0.161	2.789	0	5.767
FRY	21.597	11.374	8.921	27.964	22.238	23.961	15.247	55.34
FSY	16.412	12.51	8.177	23.644	29.667	32.482	0	51.75
Vigor	-	0.204	-	0.271	-	0.002	-	0.399
NRP	-	3.959	-	1.837	-	23.108	-	6.498

leaf retention (LR), dry root yield (DRY), dry matter content (DMC), fresh root yield (FRY), fresh shoot yield (FSY), plant vigor (Vigor), number of roots per plant (NRP).

$\sigma^2_{C\times L\times Y}$ variance component of clone, location, and year interaction; $\sigma^2_{C\times Y}$ variance component of clone and year interaction; $\sigma^2_{C\times L}$ variance component of clone, and location interaction; σ^2_{C} variance component of clone; $\sigma^2_{L\times Y}$ variance component of location and year interaction; σ^2_{Y} variance component of year; σ^2_{L} variance component of location; σ^2 variance component of residual.

Table S2. Deviance analysis for all cassava agronomic attributes.

	$\sigma^2_{C\times L\times Y}$	$\sigma^2_{C\times Y}$	$\sigma^2_{C\times L}$	σ^2_{C}	$\sigma^2_{L\times Y}$	σ^2_{Y}	σ^2_{L}
LR	**43.1**	0	0	1.06	0	2.5	0.18
DRY	**112.5**	**11.92**	0.64	**70.07**	**17.67**	0.6	0
DMC	**138.67**	**22.47**	0	**145.52**	1.99	2.58	0
FRY	**127.63**	**15.37**	**60.33**	**208.26**	**22.73**	0.94	0.13
FSY	**88.87**	**20.26**	**60.22**	**168.42**	**42.96**	2.05	0
Vigor	-	**269.61**	-	**278.04**	-	0.01	-
NRP	-	**187.47**	-	**160.84**	-	**271.67**	-

leaf retention (LR), dry root yield (DRY), dry matter content (DMC), fresh root yield (FRY), fresh shoot yield (FSY), plant vigor (Vigor), number of roots per plant (NRP).

$\sigma^2_{C\times L\times Y}$ variance component of clone, location, and year interaction; $\sigma^2_{C\times Y}$ variance component of clone and year interaction; $\sigma^2_{C\times L}$ variance component of clone, and location interaction; σ^2_{C} variance component of clone; $\sigma^2_{L\times Y}$ variance component of location and year interaction; σ^2_{Y} variance component of year; σ^2_{L} variance component of location; σ^2 variance component of residual.

Significant effects are in bold.
